# The evolution and driving mechanism of land use classification systems in China

**DOI:** 10.1038/s41598-023-48093-6

**Published:** 2023-11-24

**Authors:** Hui Xiang

**Affiliations:** College of Geography and Tourism, Hunan University of Arts and Science, Changde, 415000 Hunan China

**Keywords:** Biogeochemistry, Environmental social sciences

## Abstract

It is of great value to explore the evolution and dynamic mechanism of land use classification systems, such as improving the current classification system and providing guidance for scientific land use methodologies. In this study, the evolution process of land use classification systems in China is analyzed, the characteristics of which in terms of content-form, the urban–rural land use relationship and land functionality are compared, and the evolution mechanism of which is evaluated. The findings reveal that: (1) The land use classification systems in China have undergone a three-stage evolution process, comprising an initial "exploration stage", followed by an "improvement stage", culminating in a "maturity stage"; (2) The content and form of these systems exhibit distinct characteristics, marked by the refinement of construction land, stability in hierarchy, and an increase in the number of classifications. The urban–rural land use relationships have transitioned from a state of "urban–rural separation" to "urban–rural coordination", and ultimately to "urban–rural integration". Moreover, land functions have evolved from single to comprehensive; (3) The evolution of land use systems is primarily driven by national policies, socioeconomic development, and resource endowments, and in essence, it is constrained by the man-land relationship. To meet the needs of global village development, future land classification systems should strive to establish universal international standards.

## Introduction

Land use pertains to the utilization of land by humans, either directly or indirectly, to fulfill their survival, developmental, and environmental prerequisites^1^.

, and it is the purposeful use and protection of land resources^2,3^. Land use classification, in essence, entails categorizing land types based on their current utilization status and inherent characteristics^4^, ultimately aiming to discern the spatial compositional units of land utilization^5^. With the changes in natural conditions, socio-economic conditions, and national policies, the land use classification system is constantly evolving. It is fundamentally pivotal and holds substantial significance to explore the evolution and dynamic mechanism of land use classification systems, such as improving the current classification system^8^, refining policies governing land use^9^, providing guidance for scientific land use methodologies^10^, and facilitating sustainable exploitation of land resources^6,7^.

The academic realm has witnessed a burgeoning interest in land use classification systems. The existing land use classification systems include national, regional, and global scales^8^. Presently, several nations, including the United States, Canada, Mexico, South Africa, and China, have instituted national land use classification systems for gathering land use data, employing two primary methodologies: sampling surveys and remote sensing image analysis^8^. At the regional level (embraced by entities such as the European Union, North America, Africa, and Asia), and the global level (promulgated by the United States Geological Survey, the UNEP/FAO, among others), land use classification systems are comparatively scarcer^9^. Concurrently, remote sensing image analysis represents the principal technique for procuring land use data at regional and global scales^9^. Predominant research endeavors are directed towards land use classification systems at the regional^10,11^ and global tiers^12,13^. Developed countries are the central focus in studies pertaining to national level classification systems^14,15^, whereas the exploration of such systems in developing nations remains limited.

The evolution and underpinning motivations of land use classification systems are pivotal facets of scholarly investigation. Scholars have scrutinized the evolutionary trajectory of land use classification systems in the United States and the United Kingdom, and have assessed the challenges inherent in global land use classification systems^13^. Research within the United States and the United Kingdom has shown that flexible and scalable land use classification systems exhibit greater rationality^16^. Furthermore, the coordination of disparate land use classification systems presents a formidable challenge^13^. In the pursuit of enhancing the practicality of existing classifications, scholars have proposed several recommendations: (1) The incorporation of novel concepts and methodologies, including the classification method encompassing production, construction, and ecological land^17,18^, the categorization and management of land endowed with ecological functions^19^, urban land classification grounded in Landsat data^20,21^ and the random forest classification approach^22–24^. (2) The establishment of connections between divergent land classification systems, such as employing satellite imagery to standardize global land use classification^25,26^, harmonizing rule systems governing land use classification^27,28^, and integrating disparate land classification frameworks^28,29^. Nevertheless, the bulk of these inquiries predominantly address pragmatic concerns^26,30^, with limited theoretical analysis^31,32^. In particular, there is a dearth of scientific investigations into the factors influencing the evolution of land use classification systems and the mechanisms driving this evolution^33,34^.

As a developing nation characterized by a sizable population and finite land resources, China confronts the pressing need for judicious management and exploitation of natural resources^19^. It is concurrently undergoing a transition from land resource management to a broader spectrum of natural resource management^22–24^. Nevertheless, fewer studies have explored their improvement direction, aligning it with the requisites of unified natural resource management. In light of these considerations, this study examines the six categories of land use classification systems promulgated in China, with a focus on addressing three critical questions:

1. What characterizes the evolutionary trajectory and attributes of China's land use classification system?
2. What dynamic mechanisms underlie the evolution of China's land use classification system?
3. How can the existing land use classification system in China be ameliorated?

This paper has substantial theoretical and practical relevance. On the one hand, it endeavors to formulate a new paradigm and enrich the foundational theory of land use classification. On the other hand, it seeks to provide a valuable reference for the enhancement of land use classification, ultimately contributing to scientific governance and rational utilization of natural resources within China.

## Materials and methods

### Research objects

Since the establishment of the People's Republic of China in 1949, various land use classification systems have been promulgated by governmental departments, including those responsible for land, agriculture, and construction, in alignment with their specific operational requirements. The inaugural nationwide land survey marked a significant milestone in 1984, with the release of the "Classification and Significance of Land Use Status" by the National Agricultural Division Committee. Subsequently, over the years, these land use classification systems have undergone five revisions to cater to the demands of detailed land investigations, cadastral surveys, and assessments of land alterations. Consequently, there are six distinct land use classification systems of this nature (Table 1), which constitute the focal point of this study. For simplicity, they are denoted by the year of promulgation, as in the 1984 version.

**Table 1:** The main land use classification systems of China.

Year	Name	Units	Main application
1984	Classification and meaning of land use status	National agricultural division committee	The first national land survey
1989	Classification and meaning of urban land	Former national land management bureau	Urban land survey
2001	National land classification (trial)	Former ministry of land and resources	Land change survey, cadastral survey
2002	National land classification (applicable during the transition period)	Former ministry of land and resources	Land change survey
2007	Classification of land use status (GB/T21010-2007)	State administration for quality control and national standards committee	The second national land survey, evaluation and statistics
2017	Classification of land use status (GB/T21010-2017)	State administration for quality control and national standards committee	The third national land survey, planning and statistics

### Data

The data employed in this study comprised information pertaining to six land use classification systems and the corresponding policy documents in China. The former dataset was sourced from the Natural Resources Department of China (https://www.mnr.gov.cn/), whereas the latter was obtained from the official website of the Chinese government (https://www.gov.cn/).

### Research methods

In this study, the historical and comparative analysis methods were chosen for their simplicity and practicality. Their combination offers an objective means of elucidating the sequence of events, comprehending developmental trajectories, and unveiling inherent characteristics. Consequently, these methods were employed to delve into the historical backdrop of the six land use classification systems, examine their evolutionary traits, and scrutinize the driving mechanisms.

#### Historical analysis method

Historical analysis method involves scrutinizing historical phenomena to elucidate their contextual significance. This process hinges on comparisons and linkages between backgrounds, conditions, contradictions, stages, and specific historical and environmental attributes of distinct entities. Employing this approach, we first engaged in a comprehensive review of the pertinent literature, analyzed the shortcomings of existing research, and proposed the research concept. Secondly, we dissected disparities in land use policies, population policies, economic development, and natural resource conditions during different historical periods. This enables an indepth analysis of the historical context surrounding the promulgation of land use classification systems. Finally, we divided the land use classification system into three stages (exploration stage, improvement stage and maturity stage) based on the above analysis.

#### Comparative analysis method

The comparative analysis method entails objective juxtaposition of various facets and entities to discern connections and delineate their dynamic patterns and developmental trajectories. In this study, the comparative analysis method was used to contrast the similarities and distinctions among land use classification systems at various stages. This comparison spanned three critical aspects: content and form, urban–rural land use relationships, and land functions. In doing so, we unveiled the fundamental laws governing their evolution. In addition, we compared the differences in national policies, socio-economic development, and resource endowment at different stages, revealing the evolution driving mechanism of China's land use classification system. At last, we revealed the evolution characteristics of human-land relationships during different historical periods, to analysis the evolution essence of land use classification systems.

### Research logic

**Figure 1:**
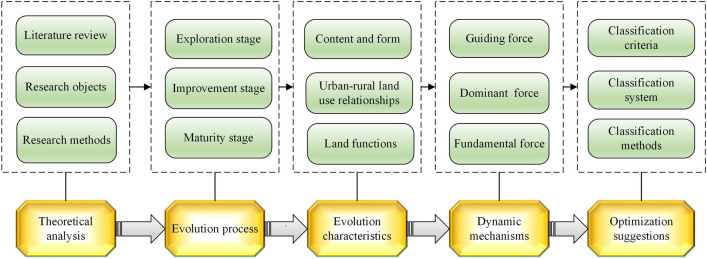
Logic diagram of the research.

The logical framework of this study encompasses theoretical analysis, evolutionary processes, evolutionary characteristics, dynamic mechanisms, and optimization suggestions, as illustrated in Fig. 1. To elucidate this framework.

The initial stages involve defining the research objects and designing the research methods, which are rooted in a comprehensive literature review.

Subsequently, an indepth analysis of the background of the six land use classification systems was conducted, and the evolutionary process was systematically explored.

Evolutionary characteristics were then compared, considering aspects such as content and form, urban–rural land use relationships, and land functions.

The driving mechanisms underlying the evolution of these systems were unveiled, considering the guiding forces, dominant forces, and fundamental forces, all within the context of the intricate man-land relationship.

Finally, this study explored revision directions within the broader context of national ecological civilization construction, economic globalization, and the development of the information industry.

## Results

### The evolution process of China's land use classification systems

In the subsequent section, we delve into the historical backdrop of the six classification systems and elucidate their hierarchical arrangements, structures, and inherent traits. Notably, the structural components entail the division of land into categories, such as agricultural land, construction land, and unused land, as outlined in accordance with the Land Management Law of China. Over time, through a sustained process of refinement and enhancement, China's land use classification systems have traversed an evolutionary trajectory, progressing from an exploratory phase to an improvement phase, and ultimately attaining a state of maturity, as summarized in Table 2.

**Table 2:** The evolution process of land use classification systems in China.

Stage	Version	Hierarchy	Structure	Characteristics
Exploration stage (1949–1990)	1984 version	Two levels (8 + 46)	48:24:28	Single application scope, few land types, simple classification, and single land function
	1989 version	Two levels (10 + 24)	16:80:4	
Improvement stage (1991–2002)	2001 version	Three levels (3 + 15 + 71)	38:45:17	Unified urban–rural land types, focus on agriculture and construction land, and highlight production and living functions
	2002 version	Three levels (3 + 10 + 52)	52:25:13	
Maturity stage (Since 2003)	2007 version	Two levels (12 + 57)	28:58:14	Unified urban–rural land types, connected multiple departments, focus on construction land, and highlight the ecological function
	2017 version	Two levels (12 + 73)	30:58:12	

#### Exploration stage (1949–1990)

Since the establishment of the People's Republic of China, the nation has experienced a significant demographic upsurge owing to lenient population policies^35^. The total population surged from 450 million in 1949 to over 980 million in 1980^36^. Commencing from 1955, the government actively encouraged urban youth to participate in rural areas while imposing stringent regulations on rural-to-urban migration^37^. In 1958, the policy emphasizing grain production as a pivotal task for promoting comprehensive development was introduced^38^. Subsequently, in 1978, the reform and opening-up policy was enacted, alongside the propagation of the Household Responsibility System in rural regions^39^. These initiatives substantially bolstered the rural population and invigorated agricultural activities. Nevertheless, challenges such as excessive occupation of arable land and haphazard land management have become increasingly salient. In this context, the Classification and Meaning of Land Use Status was formulated in 1984, aiming to comprehensively assess national land use patterns and provide guidance for agricultural production^40^. Subsequent to these developments, urban–rural interactions intensified, with urban youth returning to cities and a substantial influx of rural labor into urban areas, coinciding with the transition from a planned economy to a market-driven one. In 1986, the National Land Administration issued the Land Management Law, permitting the paid transfer of construction land. Two years later, in 1988, the revised Land Management Law allowed for land leasing. To attract foreign investment, both the central and local governments actively promoted the establishment of enterprises and development zones. These measures markedly reinvigorated urban economic development, resulting in a diversification of urban land use types. Consequently, to effectively manage the increasingly multifaceted urban land utilization, the Classification and Meaning of Urban Land was issued in 1989. Notably, the land use classification systems during this period exhibited the following distinctive features:

1. The application scope of these classification systems is limited to a single domain. At this stage, the two classification systems operate independently, with distinct criteria for urban and rural land. The agricultural sector's classification system (1984 version) primarily addresses agricultural land, tailored for application in rural areas. Conversely, the system introduced by the former National Land Management Bureau (1989 version) primarily focuses on construction land, designed for urban contexts.
2. These classification systems exhibit simplicity characterized by a reduced number of land types. During this phase, the classification systems maintain a two-tier structure with a relatively small array of land categories. Notably, the 1989 version features only 10 primary land types and 24 secondary land types. In contrast, the 1984 version provides a more intricate breakdown of agricultural land, encompassing a total of 22 secondary land categories, constituting 48% of the total. The primary level includes four fundamental types: cultivated land, garden land, forest land, and pasture land. Additionally, certain aquatic areas, such as reservoir water surfaces, pond water surfaces, and ditches, are also classified as agricultural land. Remarkably, approximately 80% of the land types in the 1989 version pertain to construction land, including seven types at the primary level, such as commercial and financial land, municipal land, construction land, and transportation land. Furthermore, the 1984 version provides a more comprehensive classification of unused land, encompassing various categories such as grassland, marshland, sandy land, bare land, bare rock gravel land, ridge land, and some aquatic areas like river water surfaces, lake water surfaces, reed land, mudflat, glacier, permanent ice, and snow-covered land.
3. The classification of land types primarily revolves around singular land functions, grounded in either utilization attributes (related to living and production functions) or production characteristics. This approach facilitates the examination and governance of land resources; however, it does not seamlessly integrate the two functions. Additionally, there is a noticeable lack of emphasis on ecological functions, which hinders the comprehensive utilization of the ecological value inherent in land resources.

#### Improvement stage (1991–2002)

The lenient population policies promulgated in the early stage has yielded a substantial demographic dividend^41^. This dividend, in turn, catalyzed rapid economic growth, with over 15% of China's economic expansion between 1982 and 2000 attributed to this demographic phenomenon^42^. Additionally, it spurred the swift expansion of urban land^43^. In 1998, the Land Management Law underwent its second revision, categorizing land into agricultural land, construction land, and unused land, while imposing strict limitations on the transfer of agricultural land. Simultaneously, in 1992, the objective of reforming the market economy system was established, propelling the development of secondary and service sectors and facilitating urban and rural exchanges. To further the objectives of a market-driven economy and enable integrated management of urban and rural land, the National Land Classification (Trial) was issued in 2001. Subsequently, the National Land Classification (Applicable during the Transition Period) was introduced in 2002, enhancing the adaptability and operability of land surveys. Owing to the incomplete national cadastral survey, areas with completed surveys adhered to the 2001 version, while unfinished areas followed the 2002 version. Consequently, urban and rural cadastral surveys were conducted separately. The land use classification systems during this period exhibited the following features:

1. The classification systems achieved a unification of urban and rural land types, facilitating standardized management of urban and rural land resources. However, they did not address the issue of coordination across multiple government departments, failing to meet the requirements of other sectors such as forestry, water conservancy, and transportation. Furthermore, regions with completed and ongoing cadastral surveys adopted differing classification standards, hindering the effective sharing of cadastral data.
2. The emphasis remained on agriculture and construction land. These land use classification systems featured a three-tier structure, with primary types comprising agricultural land, construction land, and unused land. With the exception of construction land, the categories for agricultural land and unused land remained identical. Agricultural land encompassed all land types from the 1984 version while incorporating additional categories like livestock breeding land, facility agricultural land, and rural roads. Construction land included a total of eight secondary land types, primarily aligning with those found in the 1989 version with minor adjustments. Unused land retained the classification from the 1984 version, consisting of two secondary land categories: unused land and other land.
3. These classification systems emphasized the production and living functions. The nomenclature of land types in this phase was derived from considerations of production capacity, actual use, and management characteristics, promoting unified management of urban and rural land and the sharing of survey results. However, insufficient attention was given to ecological functions, limiting their utility in ecological land resource management.

#### Maturity stage (since 2003)

With the implementation of the family planning policy and the accelerated urbanization^44^, the challenges associated with an aging population in China have emerged^45,46^. Meanwhile, the income disparity between urban and rural regions has prompted a significant migration of villagers to cities. However, due to the urban–rural dual system, these individuals often encounter difficulties integrating into urban life. Addressing the issue of farmers transitioning to urban citizens has become an urgent concern. In 2003, the Chinese government introduced the goals of "transforming the urban–rural dual system and establishing a coordinated development mechanism." Subsequently, in 2007, the Urban and Rural Planning Law was enacted, emphasizing the need to bolster the management of urban and rural land while promoting comprehensive and coordinated development between these two realms. Concurrently, the advent of "digital land" gained prominence owing to technological advancements. In 2004, the Land Management Law underwent its third revision, articulating the ambition of establishing a national land information system. In pursuit of unified urban–rural land management and cadastral informatization, the "Classification of Land Use Status" (GB/T21010-2007) was introduced. As land use intensified, ecological land issues emerged more prominently. Simultaneously, the global technological revolution fueled the rapid growth of emerging industries. In response to the management requirements of ecological land and emerging industry land, the Classification of Land Use Status (GB/T21010-2017) was promulgated. The land use classification systems during this phase exhibited the following characteristics:

1. A unification of urban and rural land types and enhanced interdepartmental connectivity were achieved. Both classification systems introduced a comparison table, establishing one-to-one or one-to-many relationships with various governmental departments. This enhanced versatility for multiple departments, including those responsible for land, forestry, agriculture, water conservancy, and transportation.
2. A heightened focus on construction land and refined land types characterized these classification systems. At this stage, the classification systems maintained a two-tier structure with 12 primary land types. The secondary land types were notably expanded, with the 2017 version featuring 73 types. Agricultural land underwent minor adjustments in forest land, with the addition of seven types, and grassland, which saw the inclusion of swamp grassland. The categorization of construction land witnessed significant growth, with most types experiencing expansion, some reduction, and a small portion reclassified into other categories. Unused land was replaced by other land and water areas, with slight adjustments such as the inclusion of other grasslands, the cancellation of reed land, and the subdivision of mudflat land, while some categories remained unchanged.
3. A pronounced emphasis on ecological function was evident in these classification systems. They centered on the ecological attributes and environmental functions of land, with the 2017 version introducing a Wetland Classification Table. This table divides wetland types, facilitating wetland statistics and management, the advancement of ecological civilization, and the development of eco-friendly land use models.

### The evolution characteristics of China's land use classification systems

**Figure 2:**
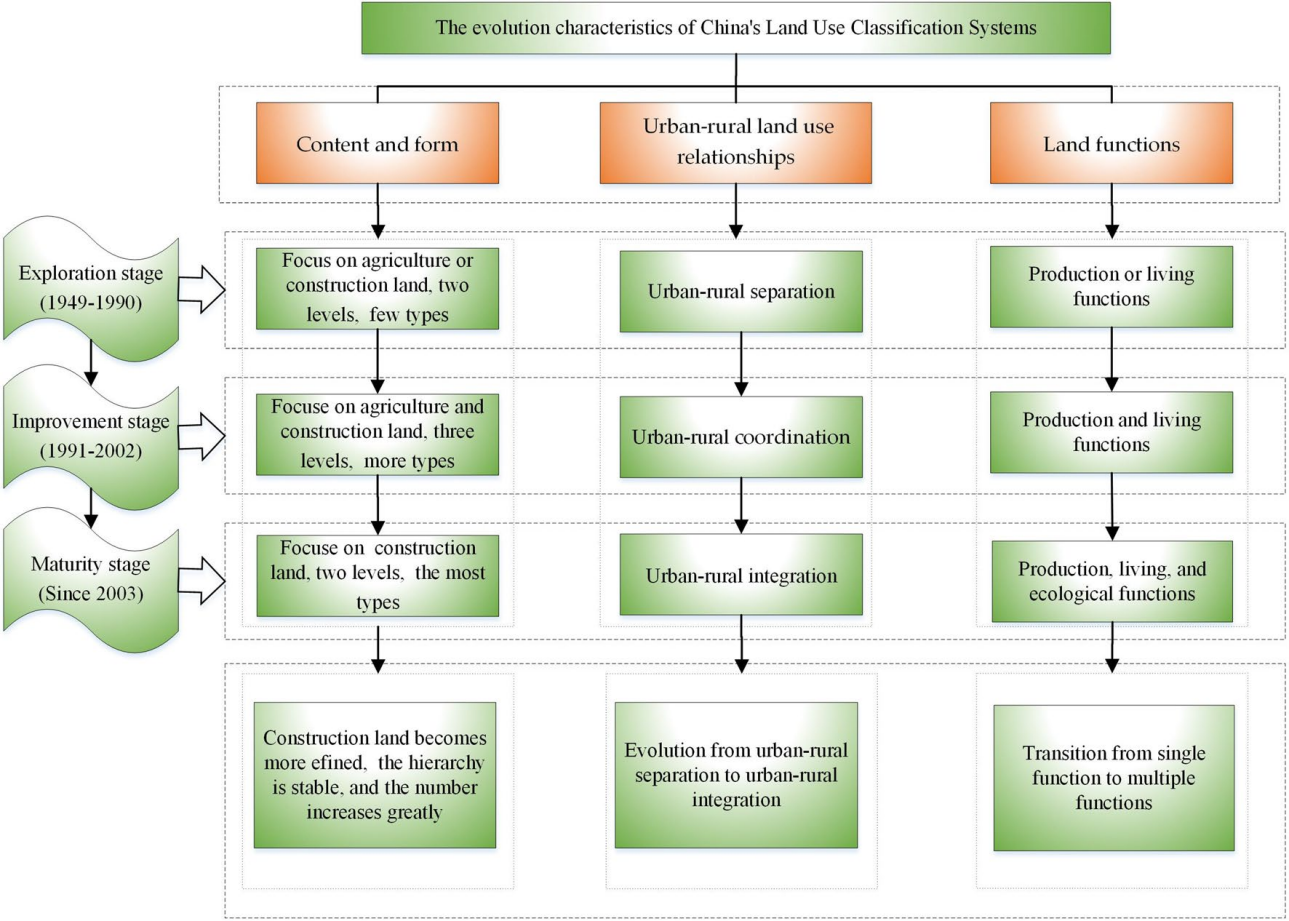
The evolution characteristics of land use classification systems in China.

The evolution of the land use classification system encompasses both explicit and implicit dimensions^31,32^. The explicit dimension is manifest in both content and form^19^, while the implicit dimension is discernible through the interplay between urban and rural land use relationships and land functions^8^. Consequently, this study undertakes an analysis of its evolutionary traits by considering three pivotal aspects: content and form, urban–rural land use relationships and land functions (Fig. 2).

#### The evolution characteristics of content and form

The evolution of land classification across these three stages has undergone a transformation from "prioritizing agriculture or construction land" to "emphasizing both agriculture and construction land" and subsequently to "prioritizing construction land". This trend also demonstrates a pattern of "limited changes in agricultural land, significant alterations in construction land, and some adjustments in unused land". Agricultural land maintains the core categories from the 1984 version, with primary-level land types remaining unchanged (including arable land, garden land, forest land, and grass-land), while secondary-level land categories undergo slight adjustments. Conversely, construction land undergoes a gradual refinement based on the 1989 version, featuring adjustments at the primary level and further subdivision at the secondary level. For instance, transportation land introduces new subcategories such as rail transit land, transportation service stations, and pipeline transport land. Unused land undergoes adjustments based on the 1984 edition, with primary-level land types being replaced by other land and water areas, while secondary-level categories experience minor changes. The classification structure maintains a stable, dendritic, and multi-level framework, characterized by fewer hierarchical levels and a greater variety of land types, indicating a move towards greater refinement. In contrast, the land use classification system in the United States consists of four levels^47^. The first and second levels are applied nationally or at the state level, while the third and fourth levels are designed for smaller areas and can be flexibly expanded^48^. The land use classification of the United Nations Food and Agriculture Organization comprises two levels^49^, expandable according to user requirements. The European Union's land use classification operates with three levels at a broad scale and allows for flexible subdivision at a smaller scale^50^. In China, most land use classification systems are composed of two levels (although some feature three levels), but none of them permit unrestricted sub-division. Furthermore, except for the initial exploration stage, these systems include more than 50 land types, with a maximum of 73 types.

#### The evolutionary characteristics of urban–rural land use relationship

The dynamic evolution of the urban–rural land use relationship demonstrates a transition from "urban–rural independence" to "urban–rural coordination", culminating in "urban–rural integration". During the initial exploration stage, separate land use classification systems were in place for urban and rural areas, with different issuing units and distinct emphases on land types. Subsequently, in the improvement stage, the land use classification systems shifted away from the independent patterns of urban and rural land types and adopted unified classification standards. Finally, in the maturity stage, these systems catered to the requirements of other specialized departments, fostering closer interdepartmental collaboration and facilitating the sharing of data resources.

#### Evolution characteristics of land functions

Land serves as a multifaceted resource, encompassing the vital functions of production, living, and ecology^51,52^, and the above. These three functions are interlinked and mutually reinforcing. Over the study period, land functions have transitioned from singular to comprehensive roles. The evolution of land use classification systems in China mirrors this progression, evolving from an emphasis on either production or livingfunctions to an integration of production, living, and ecological functions. This integration aligns with the broader ecological civilization strategy, facilitating the optimization of the ecological environment, promoting the sustainable utilization of land resources, and fostering harmonious development across the realms of economy, society, and ecology.

### The evolution dynamic mechanism of China's land use classification systems

**Figure 3:**
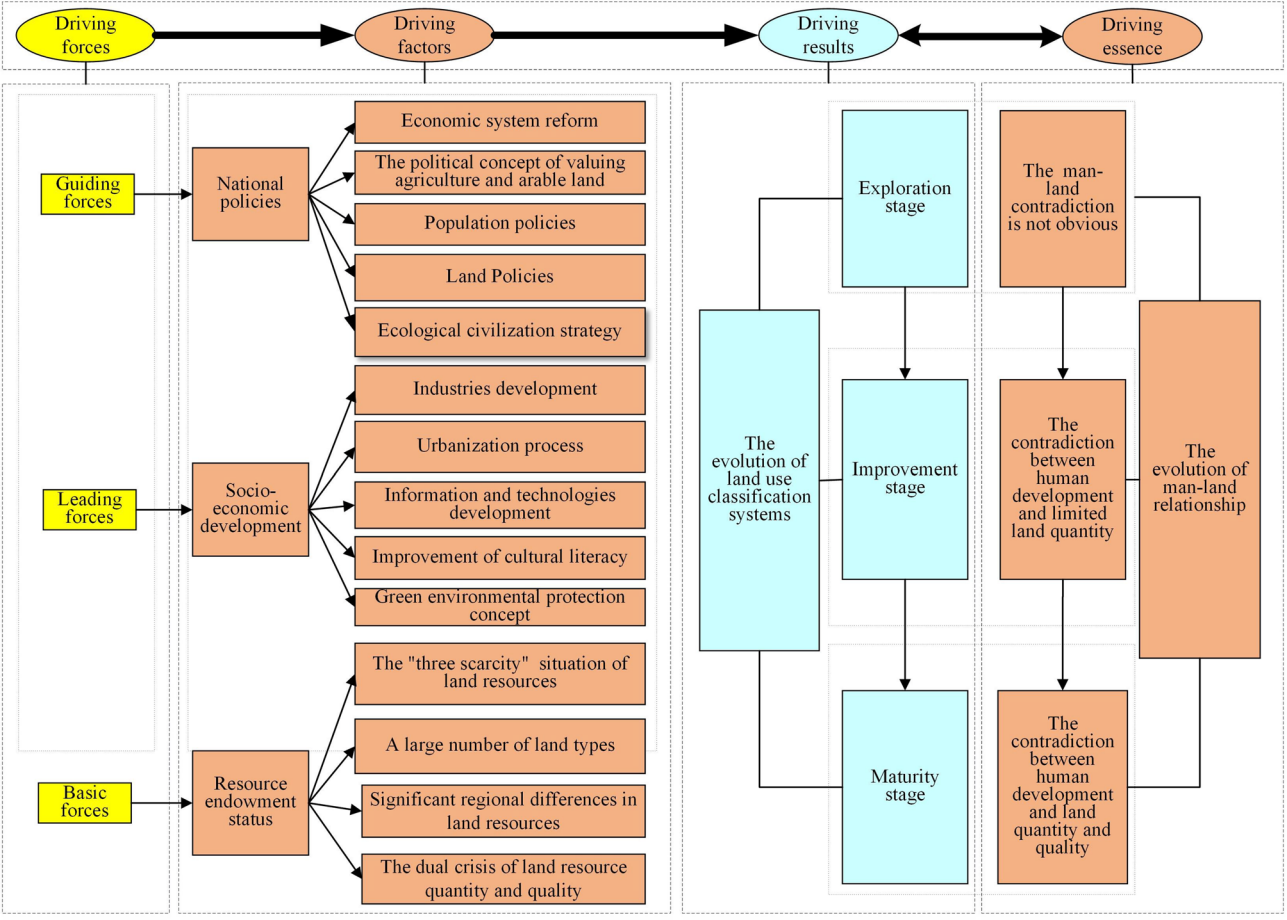
The evolution dynamic mechanism of China's land use classification systems.

The evolution of China's land use classification systems is shaped not only by national policies but also constrained by the prevailing social and economic context as well as the state of resource and environmental conditions. Consequently, a comprehensive understanding of the internal mechanisms necessitates an exploration of three key facets: national policies, socio-economic development, and the resource endowment status. This exploration is rooted in the intricate interplay between human activities and the land, as illustrated in Fig. 3.

#### National policies are guiding forces

The evolution of land use classification systems in China is intricately guided by government policies. Firstly, the dynamics of urban–rural land use relationships are influenced by economic system reforms. Following the establishment of the People's Republic of China, the planned economy system was implemented, tightly regulating the flow of rural–urban production factors, which in turn gave rise to the dual urban–rural system. In 1984, the reform of the state-owned system was initiated, leading to a shift towards a market-dependent system for the movement of production factors. In 2003, the government introduced market economy reforms, and in 2007, it set the goal of urban–rural integration. These policy shifts have profoundly impacted the urban–rural land use relationships and consequently, the evolution of land use classification systems.

Secondly, the government's political emphasis on agriculture and arable land has resulted in the detailed classification of agricultural land.

Thirdly, there exists a discernible correlation between land use classification systems and population and land policies. China's population policies have transitioned from "encouraging population growth and restricting migration" to "family planning and free migration". The promulgation and revision of the Land Management Law have introduced market-oriented principles to land resource allocation. These policies have increased human activity in urban areas while mitigating land use conflicts in rural regions. Consequently, construction land types have gradually become more refined, while agricultural land and unused land types have remained relatively stable.

Finally, the ecological civilization strategy has facilitated the integration of ecological concepts into land use classification systems. Consequently, these systems have begun to prioritize the ecological value of land.

In summary, China's land use classification systems are profoundly shaped by national policies and are designed to meet the evolving needs of land resource regulation in different periods.

#### Social-economic development are the leading forces

The evolution of land use classification is primarily driven by socio-economic changes. First, industrial development plays a pivotal role in shaping land use classification. The fewer land types in the 1980s aligned with the lower economic level and limited range of industrial activities during that period. However, as urban industries have expanded, urban land types have become more refined. Nonetheless, population and industrial concentration in urban areas have also exerted a certain influence on agriculture, resulting in relatively stable agricultural land types.

Second, urbanization is a significant factor affecting land use classification systems. From 1984 to 2017, China's urban population surged by 238.71%, while its rural population declined by 28.23%. The dual urban–rural system has historically constrained population migration, to address this issue, land use classification systems are gradually shifting from urban–rural independence to integration. Simultaneously, large-scale population migration to urban areas contributes to the diversification of urban land use patterns. However, challenges related to farmland occupation and soil contamination in rural areas are becoming increasingly pronounced, presenting new ecological management challenges for agricultural land.

Third, information technology is a crucial factor influencing the evolution of land use classification systems. Advances in communication, internet, and positioning technologies have enabled the dynamic monitoring of extensive land areas. Real-time updates of land use data have made unified land resource management feasible.

Finally, the enhancement of residents' cultural literacy is a significant driver for emphasizing the ecological attributes of land. Consequently, human activities wield substantial influence over changes in land use classification, with their evolution aimed at better adapting to human production and life in different eras.

#### Resource endowment status are the basic forces

The allocation of land resources is fundamentally determined by land resource endowment, which in turn shapes land use modes and resource regulation strategies, serving as a foundational factor in land use classification. On one hand, land use classification systems consistently prioritize agricultural land types, primarily due to the objective reality of "three scarcities"—limited per capita arable land, insufficient reserve land, and a shortage of high-quality arable land. This reality has led to the implementation of the strictest farmland protection policies and stringent controls on the expansion of construction land.

On the other hand, the rich diversity of land types and land use patterns can be attributed to the vast geographical expansion, diverse climate, complex topography, and geological conditions across China. Additionally, the escalating land resource crisis has directly contributed to increased emphasis on the ecological function of land. During the 1980s, ecological issues related to land use were relatively insignificant due to lower-intensity development. However, with intensified human intervention, challenges, such as resource scarcity and declining land quality have become increasingly evident.

Furthermore, significant regional disparities in land resources have prompted a trend towards expanding classifications. China's land resource distribution is uneven and expandable classifications can effectively highlight the regional characteristics of land resources. Consequently, land use classification systems are both influenced and constrained by prevailing resource conditions, continually evolving in response to changes in resource endowment.

In summary, the evolution of China's land use classification system is intricately intertwined with national policies, socio-economic development, and resource endowment conditions, all fundamentally rooted in the dynamics of human-land relationships. Within this context, national policies serve as guiding factors, socio-economic development is the predominant force, and resource endowment acts as the fundamental determinant. In the exploration stage, human-land contradictions are less pronounced, resulting in simpler land types with a focus on single functions. In the improvement stage, the prominence of human-land contradictions leads to more complex land types and functions. Finally, in the mature stage, human-land contradictions become particularly prominent, resulting in the most intricate land types and functions. Concurrently, human-land relationships are influenced by national policies, socio-economic development, and resource endowment conditions.

## Discussion

### The revision suggestions

The global sharing of land information and data has become a pivotal aspect of globalization^13^. Consequently, the standardization and harmonization of land use classification systems represent significant trends for the future^8^. Meanwhile, China is at a critical juncture, transitioning from rapid to high-quality development^17,18^, and ensuring ecological utilization and reasonable protection of natural resources remains a critical task^51^. Therefore, China's future land use classification system must align with the imperatives of economic globalization, information technology advancement, and resolution of ecological resource challenges.

Firstly, the establishment of a globally recognized classification standard is imperative. Advances in transportation and communication technology have ushered in the era of the "digital earth", fostering global communication and cooperation^14^. Currently, land classification standards vary across different countries and regions^8^, hindering the achievement of seamless global data sharing^9^. To address this issue, the development of a global classification system that comprehensively considers commonalities at large regional scales while accommodating characteristics unique to smaller areas is essential to eliminate barriers.

Secondly, for large regions, a classification system that focuses on comprehensive land attributes is needed. This system should be constructed based on both the natural and social attributes of land resources^51^. Research efforts should be directed towards strengthening the understanding of the fundamental principles, classification methods, and key technologies underpinning this classification system.

Thirdly, in smaller areas, there should be an exploration of a classification method based on the primary functions of land. This approach should allow for the addition of new attributes and the selection or combination of various attributes according to specific needs^52^. Such flexibility is conducive to the rational management of production, construction, and ecological land. It also underscores the ecological value and regional characteristics of land resources^51,52^.

In summary, to effectively meet the demands of the global village, the future trajectory entails the establishment of a multi-level classification system that maintains a fundamental division at large regional scales while remaining adaptable and expandable for smaller areas.

### Contributions of this study

This study has established a comprehensive research framework for investigating the evolution and dynamic mechanisms of land use classification systems, following a logical progression of "theoretical analysis, evolution process, evolution characteristics, dynamic mechanisms, and optimization suggestions." The framework exhibits clear logic and a unique perspective, thereby introducing a new paradigm for the study of land use classification systems and enhancing the theoretical underpinnings of related research. In summary, this research enriches the theory and methodologies used in similar studies and serves as a guiding resource for future research.

The evolution of land use classification systems is a complex phenomenon^6,7^. Through historical analysis and comparative assessment, this study delineates the evolution process of China's six land use classification systems into three distinct stages: exploration, improvement, and maturity. Furthermore, it examines the evolution characteristics of these systems from three key perspectives: content and form, urban–rural land use relationships, and land functions. In contrast to the existing literature, which has primarily explored individual aspects^4,6,7^, this study provides a holistic analysis encompassing the background, evolution process, and evolution characteristics of China's land use classification systems. Thus, it offers a scientifically sound, comprehensive, and systematic evaluation of these systems and serves as a valuable reference for similar research endeavors.

The study also reveals that the evolution of land use classification systems is influenced by a multitude of factors spanning political, natural, and socioeconomic dimensions. These findings align with existing research conclusions^6,7^, further confirming the scientific rigor and validity of this study.

Furthermore, the proposal of an expandable classification system, which is a novel concept within China's current classification framework, adds a unique dimension to this research. Consequently, the indicators selected in this study can serve as valuable references for similar investigations, and the suggested improvements have the potential to enhance the land use classification system, fostering unified natural resource management.

### Limitations and prospects

The factors influencing the land use classification system are complex and diverse^53^, with each factor exerting varying degrees of influence in different directions^54^. Furthermore, obtaining quantitative data for land use classification systems can be challenging^29^, especially when dealing with intangible factors such as policies that are difficult to quantify^10^. Therefore, quantifying these influencing factors is a formidable task. In this study, we primarily employed qualitative exploration to analyze these factors. Consequently, the specific directions and magnitudes of their influence remain unspecified.

Moreover, the process of standardizing and unifying the land use classification system is inherently lengthy and arduous^15^. While this article has put forth some revision suggestions for China's land use classification system, it is essential to acknowledge that the applicability of these suggestions requires practical testing. This stems from the complexity, diversity, and dynamic nature of the natural resources.

To address these challenges, future research should focus on two key areas. First, it is worthwhile to gather data through research, statistical analysis, and other methodologies, and subsequently construct an impact factor analysis model to enable quantitative evaluation. With the aid of quantitative results, targeted measures can be proposed for the management, utilization, and protection of natural resources. Second, the latest classification system must be revised and applied to specific regions to assess its applicability and scientific validity. Based on the outcomes observed in these regions, further exploration of more rational optimization measures can be undertaken.

## Conclusions

This study examines the evolutionary process, delineates the characteristics of evolution, and scrutinizes the mechanisms underlying China's land use classification system. The primary findings are as follows:

1. The evolution of China's land use classification system can be delineated into distinct stages: exploration, improvement, and maturation. In the exploration phase, the system featured fewer land categories, simple classification criteria, and a limited scope of land functions. During the improvement phase, the system diversified into more land categories, with a heightened emphasis on both production and living functions, leading to unified management of urban and rural land. During the maturation phase, the system embraced a multitude of land categories, each accommodating diverse functions and applications, making it adaptable to various governmental departments.
2. The evolutionary characteristics of China's land use classification system manifest in three key dimensions: content and form, urban–rural land utilization dynamics, and the diversification of land functions. Changes in content and form are characterized by minimal alterations in agricultural land categories, substantial modifications in construction land categories, and some adjustments in unused land categories. Concurrently, a dendritic classification structure has materialized, characterized by a limited number of hierarchical levels but a plethora of land categories. The relationship between urban and rural land utilization has evolved through a sequence of phases, encompassing "urban–rural segregation", "urban–rural coordination", and "urban–rural integration". Furthermore, land functions have transitioned from singular roles (production or living) to multifaceted roles, encompassing production, living, and ecological functions.
3. The evolution of China's land use classification system is subject to the influence of multiple factors, including national policies (exerting a guiding influence), socio-economic development (acting as a leading force), and the status of resource endowments (representing a fundamental constraint). Ultimately, this evolution is fundamentally shaped by the intricate interplay between human activities and the land itself.
4. To meet the demands of the global village, the future trajectory entails the establishment of a multi-level classification system that maintains a fundamental division at large regional scales while remaining adaptable and expandable for smaller areas. So, it should be a land classification system that can be freely expanded.

## Data Availability

The data of this research are available from the corresponding author upon reasonable request.

## References

[1] Long H, Qu Y (2018). Land use transitions and land management: A mutual feedback perspective. Land Use Pol..

[2] Bussi G (2018). Modelling the effects of climate and land-use change on the hydrochemistry and ecology of the River Wye (Wales). ScTEn.

[3] Albouy D, Ehrlich G (2018). Housing productivity and the social cost of land-use restrictions. J. Urban Econ..

[4] Yang Y, Newsam S (2010). Bag-of-visual-words and spatial extensions for land-use classification. Sigspatial Int. Conf. Adv. Geogr. Inf. Syst..

[5] Rainis R, Jaafar N (2022). GIS application in evaluating land use-land cover change and its impact on hydrological regime in Langat River Basin, Malaysia. Land Use Pol..

[6] Rozenstein O, Karnieli A (2011). Comparison of methods for land-use classification incorporating remote sensing and GIS inputs. Appl. Geogr..

[7] Webb EL, Evangelista MA, Robinson JA (2000). Digital land-use classification using space-shuttle-acquired orbital photographs: A quantitative comparison with landsat TM imagery of a coastal environment, Chanthaburi, Thailand. PgERS.

[8] Yang H, Li S, Chen J, Zhang X, Xu S (2017). The standardization and harmonization of land cover classification systems towards harmonized datasets: A review. ISPRS Int. J. Geo-Inf..

[9] Nedd R (2021). A synthesis of land use/land cover studies: Definitions, classification systems, meta-studies, challenges and knowledge gaps on a global landscape. Land.

[10] Feranec J, Hazeu G, Kosztra B, Arnold S (2016). CORINE land cover nomenclature, European Landscape Dynamics. CORINE Land Cover Data.

[11] Feranec J, Jaffrain G, Soukup T, Hazeu G (2010). Determining changes and flows in European landscapes 1990–2000 using CORINE land cover data. Appl. Geogr..

[12] Congalton RG, Gu J, Yadav K, Thenkabail P, Ozdogan M (2014). Global land cover mapping: A review and uncertainty analysis. Remote Sens..

[13] Arsanjani JJ, Tayyebi A, Vaz E (2016). GlobeLand30 as an alternative fine-scale global land cover map: Challenges, possibilities, and implications for developing countries. Habit. Int..

[14] Homer C (2020). Conterminous United States land cover change patterns 2001–2016 from the 2016 national land cover database. ISPRS J. Photogram. Remote Sens..

[15] LeClerc E, Wiersma YF (2017). Assessing post-industrial land cover change at the Pine Point Mine, NWT, Canada using multi-temporal Landsat analysis and landscape metrics. Environ. Monit. Assess..

[16] Dongjin QI, Jianyun Z (2013). Evolution for land use classification theory and practice in USA and UK. City Plann. Rev..

[17] Zhengxin JI (2023). Quantitative identification and the evolution characteristics of production-living-ecological space in the mountainous area: From the perspective of multifunctional land. J. Geogr. Sci..

[18] Bettinger P, Boston K, Siry JP, Grebner DL (2017). Geographic information and land classification in support of forest planning: ScienceDirect. For. Manag. Plan..

[19] Pokharel D (2011). An ecological land classification approach to modeling the production of forest biomass. For. Chron..

[20] Mahmoudi FT, Arabsaeedi A, Alavipanah SK (2019). Feature-level fusion of landsat 8 data and SAR texture images for urban land cover classification. J. Indian Soc. Remote Sens..

[21] Pullanagari R, Kereszturi G, Yule IJ, Ghamisi P (2017). Assessing the performance of multiple spectral–spatial features of a hyperspectral image for classification of urban land cover classes using support vector machines and artificial neural network. J. Appl. Remote Sens..

[22] Xu S, Zhao Q, Yin K, Zhang F, Yang G (2019). Combining random forest and support vector machines for object-based rural-land-cover classification using high spatial resolution imagery. J. Appl. Remote Sens..

[23] Zhang XM, He GJ, Zhang ZM, Peng Y, Long TF (2017). Spectral-spatial multi-feature classification of remote sensing big data based on a random forest classifier for land cover mapping. Cluster Comput..

[24] Rodriguez VF, Ghimire B, Rogan J, Chicamo M, Sanchez JP (2012). An assessment of the effectiveness of a random forest classifier for land-cover classification. ISPRS J. Photogram. Remote Sens..

[25] Genuit K (2010). Need for standardization of psychoacoustics. J. Acoust. Soc. Am..

[26] Nsor ME, Akamigbo FOR (2009). Characterization, classification and land use management of flood plain soils of central Cross River State, Nigeria. Glob. J. Agric. Sci..

[27] Peng Y (2022). Introducing improved transformer to land cover classification using multispectral LiDAR point clouds. Remote Sens..

[28] Deb SK, Nathr RK (2012). Land use/cover classification: An introduction review and comparison. Glob. J. Res. Eng..

[29] Peng Y (2022). Introducing improved transformer to land cover classification using multispectral LiDAR point clouds. Remote Sens..

[30] Szarek-Iwaniuk P (2021). Land use capability classification adaptation in low and intermediate technology farming systems: A soil erosion indicator. Sustainability.

[31] Nedd R (2021). A synthesis of land use/land cover studies: Definitions, classification systems, meta-studies, challenges and knowledge gaps on a global landscape. Land.

[32] Keshtkar AR, Keshtkar H (2008). A land covers classification system for environment assessment in semi-arid regions of Iran. Desert.

[33] Chakraborty S, Li B, Weindorf DC, Deb S, Acree A, De P, Panda P (2019). Use of portable X-ray fluorescence spectrometry for classifying soils from different land use land cover systems in India. Geoderma Int. J. Soil Sci..

[34] Feng CC, Flewelling DM (2004). Assessment of semantic similarity between land use/land cover classification systems. Comput. Environ. Urban Syst..

[35] Wu C (2021). Mapping multi-temporal population distribution in China from 1985 to 2010 using landsat images via deep learning. Remote Sens..

[36] Liu X, Wang J, Christakos G, Liao Y (2019). China population distributions at multiple geographical scales and their correlates. J. Environ. Inform..

[37] Cheng Y, Jia S, Meng H (2022). Fiscal policy choices of local governments in China: Land finance or local government debt?. Int. Rev. Econ. Finance.

[38] Liu Y, Zhou Y (2021). Reflections on China's food security and land use policy under rapid urbanization. Land Use Pol..

[39] Haisheng HU, Dong W, Hsu CL, Pan JN (2020). How will the land revenue policy reform affect China's economy? A simulation analysis based on general Equilibrium. Sing. Econ. Rev..

[40] Su B, Li Y, Li L, Wang Y (2018). How does nonfarm employment stability influence farmers' farmland transfer decisions? Implications for China's land use policy. Land Use Pol..

[41] Liu Z, Fang Y, Ma L (2022). A study on the impact of population age structure change on economic growth in China. Sustainability.

[42] Whalley J, Zhao X (2013). The contribution of human capital of human capital to China's economic growth. China Econ. Pol. Rev..

[43] Yang Z (2009). Urban public pension, replacement rates and population growth rate in China. Ins. Math. Econ..

[44] Zhan P, Ma X, Li S (2021). Migration, population aging, and income inequality in China. J. Asian Econ..

[45] Zhan HJ (2013). Population aging and long-term care in China. Generations.

[46] Xi H (2012). Researching about the outdoor space design adjusting to aging population in south-central in China. Open J. Civil Eng..

[47] Xie Y, Lark TJ, Brown JF, Gibbs HK (2019). Mapping irrigated cropland extent across the conterminous United States at 30 m resolution using a semi-automatic training approach on Google Earth Engine. JPRS.

[48] Gao Z, Deng LX (2003). Spatial features of land use/land cover change in the United States. J. Geogr. Sci..

[49] Yang H, He Q (2019). The development characteristics of local county urban-rural land classification system and its experience in the USA. Urban Plan. Int..

[50] Latham JS, He C, Alinovi L, Digregorio A, Kalensky Z (2002). FAO methodologies for land cover classification and mapping. Springer.

[51] Fan X, Liu J, Chen J, Zhao L, Hong W (2019). Changes of land use and functions of ecosystem service: A case study in China. Pol. J. Environ. Stud..

[52] Guo W (2016). Assessment of comprehensive carrying capacity of land resources based on land functions. Asian Agric. Res..

[53] Demers MN (2014). Land classification research: A retrospective and agenda. Int. J. Appl. Geospat. Res..

[54] Jiang G, Ma W (2017). Agglomeration or dispersion? Industrial land-use pattern and its impacts in rural areas from China's township and village enterprises perspective. J. Clean Prod..

